# Genomic screening and genomic diagnostic testing—two very different kettles of fish

**DOI:** 10.1186/s13073-019-0696-9

**Published:** 2019-11-27

**Authors:** Leslie G. Biesecker

**Affiliations:** National Human Genome Research Institute, National Institutes of Health, South Drive, Bethesda, MD 20892 USA

## Editorial summary

Genomic testing can be misunderstood as being determinative, when in reality it is the same as all other tests and context is essential for its correct interpretation. Two hypothetical cases of testing for Marfan syndrome demonstrate how clinicians should contextualize genomic test results and the implementation of Bayes theorem in clinical decision-making.

Genome and exome sequencing (GS/ES) are rapidly becoming more widely used and providing unprecedented ability to diagnose individuals with rare or unexpected genetic disorders quickly and accurately. The power of these sequencing techniques is in their breadth and hypothesis-generating nature: they test for nearly all Mendelian disorders [1]. GS/ES is a powerful diagnostic tool, but like any other clinical test, it has true positives, true negatives, false positives, and false negatives. It is essential to understand these attributes both in the diagnostic setting and in the screening setting. The key to understanding variant pathogenicity and how to contextualize the clinical implications is based on Bayes theorem. Here, using two hypothetical GS/ES testing scenarios the practical utility of Bayes in genomic testing will be illustrated.

A young man presents to his internist for a routine checkup and the clinician notes that he has facial and skeletal features of Marfan syndrome that do not reach the threshold for major diagnostic specificity. He also has a history of high myopia but no known lens dislocation. There is no family history of Marfan syndrome, but several of his maternal relatives are tall with a vague history of unexplained sudden death in one. The internist sends the young man for an echocardiogram, which shows an aortic root diameter to body surface area ratio that is just over the 95th centile. On the basis of this evidence, she estimates that there is about a 75% chance that the patient may have Marfan syndrome: there are some signs of the disorder, but not enough for clinical diagnosis. Genome sequencing is ordered and returns a pathogenic variant in *FBN1* (pathogenic is defined as ≥ 99% likely to be associated with the disease).

**Fig. 1:**
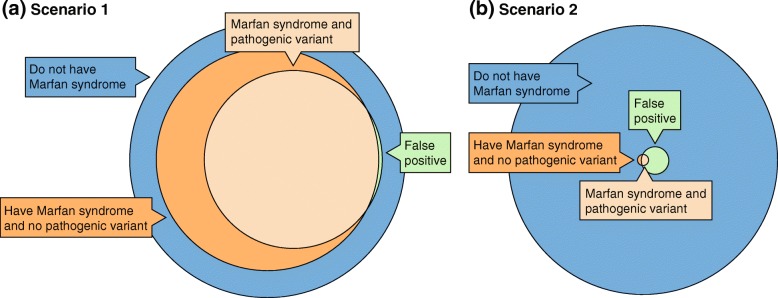
Genomic test results for patients in diagnostic and screening contexts. **a** Patients in scenario 1, in which genomic testing is used for diagnosis to support clinical signs that suggest Marfan syndrome. The overlapping *circles* represent the relative likelihood or probabilities for the scenario. The *blue circle* is all patients who have clinical signs that cause their doctors to request genomic tests. The 75% of these patients who actually have Marfan syndrome are the *dark orange circle* and patients with a pathogenic variant are the *lighter orange circle*. The small *green sliver* on the *right* are the patients who do not have the disorder but have a false-positive test—a pathogenic variant that isn’t actually causative. **b** In scenario 2, in which genomic testing is used for screening of patients without clinical signs of Marfan syndrome, the *green area* is still relatively small compared to the *blue circle*, because the false-positive rate is unchanged. What have changed dramatically are the *dark orange circle* (because the presence of the disorder is less likely in a screening scenario) and the ratio of the *green area* to the *light orange area*: in this case, a false-positive test is more likely than a correct diagnosis of Marfan syndrome

In patients with known Marfan syndrome, a pathogenic variant is identified about 70% of the time. If one screens people without Marfan syndrome, you can expect to get a false-positive result of a pathogenic *FBN1* variant about 0.1% of the time. So the test has good sensitivity and specificity. Intuitively, the internist concludes that the presence of the variant confirms the diagnosis. What is the basis for this conclusion? This is Bayesian reasoning, which takes into account what she knows before the new piece of evidence or data were acquired and then asks how that prior knowledge is either made more or less likely by the new information. Just as Bayes theorem has been used to formalize the pathogenicity assessment of the genomic variant itself [2], the same theorem can be used to make an integrated clinical assessment of the patient. The formula for the theorem is widely available but not reproduced here. This is shown graphically in Fig. 1a, where the light orange circle with its small green sliver represents the conclusion that was reached by the internist. For patients in this scenario, the likelihood that a patient has the disease is increased to a very high probability when the variant is found to be present by GS/ES. If calculated formally, the likelihood that the young man has Marfan syndrome is 99.95%. The calculation is as follows:

$$ \frac{0.75\ast 0.70}{\left(0.75\ast 0.70\right)+\left(0.25\ast 0.001\right)}=0.9995 $$

Importantly, the likelihood that the patient has the disorder (here 99.95%) is not numerically equivalent to the probability of pathogenicity of the variant (which is ≥ 99%).

In a different patient scenario that uses the same test result and test performance characteristics, a pediatrician orders GS/ES on a toddler because she has autism. No variant for the autism is identified, but there is a secondary finding of a pathogenic variant (≥ 99% pathogenicity, as above) in *FBN1*. The American College of Medical Genetics (ACMG) recommends that secondary (formerly incidental) genomic findings should be assessed in those who are found to have an *FBN1* variant, because such secondary findings can identify occult disease that is highly actionable [3, 4]. This toddler has no apparent features of Marfan syndrome and she is adopted, so she has no known family history. As part of her autism workup, she had an echocardiogram and ophthalmology evaluation, both of which were normal. Here, the outcome is very different because the genome is being used as a screening test, not a diagnostic test. Figure 1b, using the same test performance characteristics, shows that the true-positive rate for this patient is lower than the false-positive rate. The likelihood that the patient has Marfan syndrome is low on an absolute scale (~ 8.5%), but it is more than 600 times the relative risk of the general population, a huge relative risk. The calculation is as follows:

$$ \frac{0.00013\ast 0.70}{\left(0.00013\ast 0.70\right)+\left(\left(1-0.00013\right)\ast 0.001\right)}=0.085 $$

(Note here that 0.00013 is the overall prevalence of Marfan syndrome, about 1/7500.) On the basis of what is known at this point, the odds are that this toddler does not have Marfan syndrome. The dramatic change here is due to the prior probability, which was 75% in the first scenario but about 1/7500 in the second scenario. Like all tests, GS/ES is challenged by the false-positive rate, which in these scenarios is the likelihood that a pathogenic variant might not actually be causative of disease. This is implicit in the description that it is ≥ 99% likely to be causative, not 100%. The critical lesson from scenario 2 is that the prior probability of disease (1/7500 vs 75%, screening vs diagnostic) is a critical determinant of the likelihood of the diagnosis.

While it is most likely that this toddler does not have Marfan syndrome, one should not dismiss the diagnosis. There are low risks of serious medical complications of Marfan syndrome in young children, so it is reasonable for the pediatrician to recheck some of the physical findings for Marfan syndrome and, if these features are absent, adopt a watch and wait approach. He could continue with regular pediatric well checks and when the girl is older, and clinically reassess and update the interpretation of the variant. Genetics knowledge is improving rapidly and a great deal will be learned in the coming years. If the variant is still considered to be pathogenic, a more thorough clinical evaluation for Marfan should be undertaken. This could include a referral to a clinician who is experienced and confident of their skills with Marfan syndrome, an ophthalmologic evaluation to assess ectopia lentis specifically, and an echocardiogram. This suite of findings can be evaluated by a clinician expert in Marfan syndrome to determine whether further workup is required, whether a diagnosis can be made and management instituted, or whether the family can be reassured that there is no sign of the disorder and a further watch and wait approach is appropriate.

These examples estimate the likelihood that the individual actually has the diagnosis, based on what was known clinically before the test and after a GS/ES test result. There are many more factors to consider in genomic diagnosis; for example, penetrance (the likelihood the patient has manifestations of the disease if they have the disease) has to be taken into account. Marfan syndrome has very high penetrance, although a number of the manifestations are age-dependent [5]. Thus, the absence of obvious signs of the disorder in the toddler (scenario 2) should not permit the pediatrician to dismiss the possibility that signs could develop over the coming years (age-dependent penetrance). It should also be noted that calculations such as these are more complex when a disorder has low penetrance. Although there are nuances and complexities, the conclusion is clear: GS/ES results must be contextualized in a Bayesian framework to be valid clinically.

In the end, genomic testing is more similar to, than it is different from, a hematocrit or serum sodium test result. All three tests are extremely useful if interpreted correctly, given the clinical context in which they are used. The critical concepts to recognize are that the pathogenicity of the variant is not the likelihood that the patient has the disease, any more than the accuracy of a hemoglobin result is the likelihood that the patient has anemia. The clinical context in which the testing was done is a major determinant of the diagnosis of the patient. Much of the confusion surrounding genomic testing is based on misconceptions of genetic determinism: that one can determine ones’ status with certainty on the basis of a genomic or genetic test result. Genetic testing can be powerful and useful in both of the scenarios described above, but Bayes theorem must be taken into consideration.

## Conclusions and future directions

Bayes theorem applies to everything that clinicians do, whether assessing the clinical significance of a fever or that of a GS/ES result. Bayes theorem is how clinical context can be incorporated into genomic testing to allow rational clinical decision-making. By contextualizing genomic test results, clinicians can better manage their patients in both diagnostic and screening contexts.

## Abbreviation

GS/ES: Genome and exome sequencing
